# The Link Between Emotional Regulation and Impulsivity in Childhood Anxiety Disorder

**DOI:** 10.3390/children13030439

**Published:** 2026-03-23

**Authors:** Duygu Karagöz, Ece Tezsezen, Nilfer Şahin

**Affiliations:** Child and Adolescent Psychiatry Departmant, Faculty of Medicine, Muğla Sıtkı Koçman University, Muğla 48000, Turkey; ecetezsezen@mu.edu.tr (E.T.);

**Keywords:** anxiety disorders, generalized anxiety disorder, child, impulsivity, emotional regulation, anxiety sensitivity

## Abstract

**Highlights:**

**What are the main findings?**

- In generalized anxiety disorder and other anxiety disorders, significant differences were observed in emotion regulation compared to the healthy group, while no significant differences were observed in impulsivity.
- Anxiety sensitivity did not mediate the relationship between emotion regulation and impulsivity in anxiety disorders; a direct relationship between emotion regulation and impulsivity was observed.

**What is the implication of the main finding?**

- Children who have been diagnosed with anxiety disorders may display inappropriate, impulsive coping behaviors in response to emotional regulation difficulties.
- Addressing emotional regulation skills in treatment may be important for behavioral control in anxiety disorders.

**Abstract:**

**Background and Objectives:** The aim of this study is to evaluate impulsivity in childhood anxiety disorders and to examine its relationship with anxiety sensitivity and emotion regulation. **Materials and Methods:** The study group consisted of a total of 60 children aged 8–12 years diagnosed with generalized anxiety disorder (GAD, *n* = 30) and other anxiety disorders (*n* = 30). The control group consisted of 40 healthy children of similar age without a psychiatric diagnosis. Data collection forms included the Barratt Impulsiveness Scale Short Form (BIS-S), the Children’s Anxiety Sensitivity Index (ASI-3), the Emotion Regulation Checklist (ERC), and The Screen for Child Anxiety Related Emotional Disorders (SCARED)**. Results:** Our study found no significant differences in BIS-S scores between GAD, other anxiety disorders, and the control group. The total/physical and ERC subscales of the ASI-3 were higher in the generalized anxiety disorder and other anxiety disorder group than in the control group. However, there were no significant differences in the social dimension and cognitive dimension scores of the ASI-3. It has been determined that anxiety sensitivity does not significantly mediate the relationship between emotion regulation and impulsivity, and that emotional variability/negativity is directly and completely related to impulsivity. **Conclusions:** Our study suggests that children with anxiety disorders experience greater difficulties in regulating their emotions compared to healthy children, and that emotional variability is directly related to impulsivity. In this context, enhancing emotion regulation skills in anxiety disorders may prove to be a pivotal factor in the efficacy of treatment and the maintenance of behavioral control.

## 1. Introduction

The prevalence of childhood anxiety disorders is reported to be between 5.7% and 12.8% [1,2]. While studies indicate that the prevalence of anxiety disorders is comparable among boys and girls prior to adolescence, a heightened incidence of these disorders has been observed among girls following this period [3,4]. It is important to note that a complex interaction of genetic, hormonal, neurobiological, and transdiagnostic factors may play a role in the etiology of gender differences that emerge during adolescence [5]. One of the underlying mechanisms of gender differences is hypothesized to be the developmental delays in the circadian rhythm that occur during adolescence and the structural changes in sleep, which directly affect mood and emotional control [6]. Research indicates that the prevalence of anxiety-related sleep disorders is twice as high in adolescent girls compared to boys. It has been observed that hormonal fluctuations during adolescence in girls can lead to changes in sleep patterns, which may contribute to increased anxiety [7]. A longitudinal study also demonstrated that persistent anxiety and poor emotion regulation skills in adolescent girls directly mediate irritability symptoms that may serve as precursors to future anxiety and depressive disorders [8]. Current data elucidates the changes in female puberty and also demonstrates the interaction between biological and psychological mechanisms.

Genetic and neuroimaging studies are particularly salient in the biological model of the etiology of anxiety disorders [9]. According to the cognitive-behavioral model, anxiety disorders are said to have three components: cognitive, behavioral, and physiological [10]. Transdiagnostic factors, such as anxiety sensitivity and emotion regulation, which include cognitive, physiological, and behavioral components, have been demonstrated to play important roles in the development and maintenance of anxiety disorders [11,12,13]. Anxiety sensitivity, or "fear of fear," is thought to be linked to anxiety disorders through catastrophizing and misinterpretation via cognitive structures [14]. According to the emotional dysregulation model of anxiety disorders, individuals experience more negative emotions and have weak regulatory control over their emotional states [15]. It is also noted that there are gender differences in anxiety sensitivity and emotion regulation skills. Research has indicated that anxiety sensitivity is influenced by genetic, hormonal, and environmental factors in women [16], while in men it is influenced by environmental factors [17]. Research has identified discrepancies between men and women in the utilization of emotion regulation strategies [18,19]. A recent study indicated that poor emotion regulation in men predicts an increase in future anxiety sensitivity [18]. However, there is limited understanding of the relationship between anxiety sensitivity and emotion regulation in anxiety disorders. Research indicates that these factors may interact despite their classification as distinct transdiagnostic elements [20,21].

Recent studies have indicated that intolerance of uncertainty may be a transdiagnostic factor applicable across anxiety disorders [22]. In situations where the future is unclear, children with high anxiety levels tend to predict a greater prevalence of negative emotions compared to those with low anxiety levels. This tendency has been observed to diminish their coping abilities [23,24]. Based on this, transdiagnostic factors, such as emotion regulation and anxiety sensitivity, that are associated with anxiety disorders may interact in complex ways with impulsivity. Consequently, there is increasing evidence highlighting transdiagnostic biological and behavioral markers in anxiety disorders, which supports dimensional models of anxiety [25].

Impulsivity is defined as acting suddenly and without planning in response to a harmful impulse or desire despite potential negative consequences. Impulsivity is a diagnostic criterion for many psychiatric disorders and is often observed during the clinical course of these disorders [26]. Individuals with anxiety disorders actively avoid taking risks. However, this characteristic does not correspond with the fundamental characteristics of impulsivity: novelty seeking, risk taking, and acting without thinking. While some studies evaluating the relationship between impulsivity and anxiety report a negative correlation [27], other studies report positive correlations [28,29,30]. Research indicates that individuals with high anxiety levels may exhibit impulsive behavior with negative urgency when faced with situations requiring decision-making. [31,32]. Negative urgency is defined as the tendency to act impulsively in order to avoid negative emotions. It is also important to note that negative urgency, one of the dimensions of impulsivity, may be related to the impulsivity observed in anxiety disorders [33,34]. 

The relationship between anxiety sensitivity and impulsive behavior is not yet fully understood [35]. Some studies suggest that anxiety sensitivity may reduce risky behaviors in the face of potential adversity [36,37] and protect against impulsive behaviors [36]. However, other studies indicate no relationship between the two [38]. It is also important to understand the connection between emotion regulation and impulsivity in children with anxiety disorders. Emotion regulation refers to the adaptive skills that influence the processes of recognizing, experiencing, accepting, expressing, and coping with negative emotions. It has been noted that children with anxiety disorders have difficulty regulating their emotions and become irritable more easily [39,40,41,42,43]. Irritability is a well-documented symptom of anxiety disorders. It is also important to note that irritability may be a deficiency and component of emotional regulation [44]. Research indicates that girls with high irritability exhibit significantly higher levels of mood symptoms and greater challenges with impulse control compared to girls with lower irritability [8]. Given these difficulties, it has been reported that a lack of effective emotion regulation skills may be a specific risk factor in the development and persistence of impulsive behaviors [45,46,47]. A review of current data reveals a paucity of clarity regarding the interaction between anxiety sensitivity and emotion regulation difficulties with clinical symptoms, such as impulsivity, in childhood, within the framework of a conceptual model.

Research has indicated that individuals diagnosed with generalized anxiety disorder encounter heightened challenges with transdiagnostic factors, such as emotion regulation and intolerance of uncertainty, in comparison to those diagnosed with other anxiety disorders. [48,49,50,51,52]. Individuals diagnosed with GAD are reported to experience emotions with greater intensity, have difficulty distinguishing between emotions, lack strategies for regulating their emotions, and may develop inappropriate responses [15]. At this point, the anxiety sensitivity created by chronic, intense negative emotion may have a mediating effect in the relationship between emotion regulation and impulsivity by creating a negative urgency. The extant literature suggests that the relationship between impulsivity and anxiety disorders has been the subject of fewer studies in childhood than in other psychiatric disorders. Furthermore, studies in this field are often conducted in the adult age group [38,53,54,55]. Our study aimed to evaluate impulsivity, which has not yet been sufficiently clarified in childhood anxiety disorders, in both generalized anxiety disorder (GAD) and other anxiety disorders and compare it with a healthy control group. Additionally, the relationship between impulsivity in children and anxiety sensitivity and emotional regulation difficulties has been examined. Based on this, a hypothesis has been developed within a conceptual model framework that emotion regulation and impulsivity in anxiety disorders may be related either directly or through the mediating effect of anxiety sensitivity. The objective of this study is to propose an additional transdiagnostic model for conceptualizing anxiety disorders by evaluating a possible mediating factor in the etiology of anxiety disorders. Considering that impulsivity can lead to destructive behaviors, identifying possible mechanisms may contribute to the development of strategies that could affect functionality and treatment in anxiety disorders.

## 2. Materials and Methods

### 2.1. Study Design, Population, and Data Collection

A total of 60 children, aged 8–12, were identified as the study group based on psychiatric evaluation and clinical observation. The group included those diagnosed with GAD (n = 30) and those diagnosed with other anxiety disorders (n = 30). The diagnostic evaluation was performed according to the Diagnostic and Statistical Manual of Mental Disorders, 5th edition (DSM-V), and The Schedule for Affective Disorders and Schizophrenia for School-Age Children—Present and Lifetime Version (KIDDIE-SADS-PL).

The study group consists of children diagnosed with Anxiety Disorder who are undergoing outpatient treatment at our clinic, who are not receiving psychotropic medication, and who do not have any additional psychiatric diagnoses other than Anxiety Disorder, as well as no known genetic, neurological, or metabolic disease diagnoses. Cases with additional psychiatric disorders and cases receiving psychotropic medication were excluded from the study due to the frequent presence of impulsivity as a symptom in psychiatric disorders and the potential effect of psychotropic medications on impulsivity.

The control group consists of individuals who visited our outpatient clinic for counseling due to divorce, sibling rivalry, behavioral issues, peer relationships, etc. These individuals were found not to have any psychiatric disorders according to the evaluation conducted by a child psychiatrist and the KIDDIE-SADS-PL. Furthermore, these individuals did not have any known genetic/neurological/metabolic disease diagnoses. The group under consideration consisted of 40 children aged 8–12. All participants were evaluated by a child psychiatrist according to the DSM-V diagnostic criteria. The exclusion of adolescents from the study was due to the recognition that impulsivity can also be considered a characteristic symptom of adolescence. A comprehensive psychiatric evaluation was conducted on all subjects, revealing no diagnosis of mental disability. The subjects demonstrated no clinical indications of intelligence, had acquired reading and writing skills in a timely manner, and exhibited academic aptitudes commensurate with their age and grade level. The researcher collected sociodemographic information on all subjects using a form that he had prepared.

All children in the experimental and control groups were given the Childhood Anxiety Screening Scale, the Barratt Impulsivity Scale Short Form, and the Anxiety Sensitivity Index for Children. They were also instructed on how to complete the scales. The children completed the scales. The Childhood Anxiety Screening Scale was used to evaluate the severity of anxiety disorders in the study group. Mothers were asked to complete the Emotion Regulation Scale to evaluate their children’s ability to regulate their emotions.

### 2.2. Data Collection Tools

#### 2.2.1. Sociodemographic Data Form

Created by clinicians, this form collects information about the sociodemographic characteristics of children and their families. The form contains questions designed to determine the child’s age and gender, as well as the parents’ age, educational status, and socioeconomic level.

#### 2.2.2. The Schedule for Affective Disorders and Schizophrenia for School-Age Children (Present and Lifetime Version, (KIDDIE-SADS-PL)

This is a semi-structured interview that was developed in accordance with the diagnostic criteria of the DSM-V to investigate the current and lifetime psychopathology of children between the ages of 6 and 18. Ref. [56] conducted the Turkish validity and reliability study of the scale developed and organized [57].

#### 2.2.3. Emotion Regulation Checklist (ERC)

Developed by Shields and Cicchetti (1997) [58], this checklist is used to assess emotion regulation competence in children aged 6–13. Kapçı et al. established the Turkish validity and reliability of the ERC, which is used to assess children’s emotion regulation skills [59]. The ERC consists of two subscales: Emotional Variability/Negativity and Emotion Regulation. Parents and teachers who know the child can complete the scale. In our study, it was completed by mothers.

The Emotional Variability/Negativity subscale has 15 items, and the Emotion Regulation subscale has nine items, for a total of 24. The options “never, rarely, often, and always” are scored as 1, 2, 3, and 4, respectively, using a four-point Likert scale. The first factor, “emotional variability/negativity,” includes statements related to emotional instability, anger outbursts, mood swings, emotional intensity, and an inability to regulate positive emotions. A high score on this subscale indicates that a child has difficulty regulating their emotions. The Emotion Regulation subscale, on the other hand, includes factors related to adaptive emotion regulation, such as understanding emotions, empathy, emotional awareness, and prudence. A high score on the Emotion Regulation subscale indicates that the child is good at regulating their emotions.

#### 2.2.4. The Screen for Child Anxiety Related Emotional Disorders (SCARED) Child Form

The Turkish validity and reliability study of the SCARED, which was developed by Birmaher and colleagues in 1997 [60] was conducted by Çakmakçı et al. [61]. Both a parent and child form are available. The child form was used in our study. It contains 41 items that assess the child’s anxiety. Each item is scored 0, 1, or 2 based on symptom severity, yielding a total score and five factor scores. A total score of 25 or higher indicates the presence of an anxiety disorder.

#### 2.2.5. Anxiety Sensitivity Index-3 (ASI-3)

Developed by Silverman et al. (1991) [62] by adapting the Anxiety Sensitivity Index for children, the ASI-3’s validity and reliability were established by Savaş et al. [63]. This self-report scale inquires about emotions experienced in response to internal and external stimuli that may cause anxiety in children. It is recommended for use in comparative studies. The scale consists of 18 items organized into three main categories: physical, social, and cognitive. The scale is organized as a 3-point Likert scale, scored as never (1), somewhat (2), or very much (3). The minimum score is 18, and the maximum is 54.

#### 2.2.6. Barratt Impulsiveness Scale-Short Form (BIS-S)

The BIS-S is a unidimensional, eight-item self-report scale derived from the BIS-11 scale developed by Steinberg and colleagues [64]. The BIS-S is a self-report scale used to assess impulsivity. Its validity and reliability in Turkish have been established by Güleç et al. [65]. Respondents rate the presence of the symptoms described in each item on a four-point Likert scale ranging from “never” to “almost always/always.” The scale has no cutoff score. A higher total score indicates a higher level of impulsivity.

### 2.3. Statistical Analysis

The study data were analyzed using the Statistical Package for the Social Sciences (SPSS, IBM Corp., Armonk, NY, USA), version 27.0. Certain sociodemographic and clinical categorical variables belonging to the study and control groups were evaluated using numbers and percentages. The chi-square test was used to compare the classified categorical variables. The Shapiro–Wilk test was used to assess normality. Variables showing a normal distribution were presented as mean ± standard deviation, while those not showing a normal distribution were presented as median (25th–75th percentile). For the comparison of the three groups, one-way analysis of variance (ANOVA) was used for normally distributed data, and the Kruskal–Wallis H test was used for non-normally distributed data. Where ANOVA revealed significant differences, Tukey or Tamhane post hoc tests were used depending on the homogeneity of variances. A significance value of *p* < 0.05 was accepted in the analyses. Pearson and Spearman correlation tests were used to evaluate the relationship between variables. Multiple comparisons were controlled within each diagnostic group using the Benjamini–Hochberg false discovery rate (FDR) procedure (m = 22 tests per group); q < 0.05 was considered significant. To address potential sociodemographic confounding, group comparisons of scale scores were additionally adjusted for maternal education, family income, and maternal employment status using ANCOVA with HC3 robust standard errors; outcomes summarized as medians were examined using quantile regression (q = 0.5) To formally test the hypothesized indirect mechanism, three mediation models were conducted within the clinical sample (n = 60) using bootstrap confidence intervals (5000 resamples).

Post hoc sensitivity analyses were conducted for the key group comparisons. With the current sample size, the minimum detectable effect for the three-group BIS-S comparison at 80% power corresponds to approximately a medium effect (f ≈ 0.32, α = 0.05), suggesting that small-to-moderate differences in impulsivity may have been missed; therefore, null BIS-S findings should be interpreted cautiously due to potential Type II error. In contrast, based on the observed three-group differences, achieved power was high (>0.98) for SCARED, ASI-3 total/physical, and ERC subscales (α = 0.05).

## 3. Results

When the groups were compared in terms of sociodemographic characteristics, it was found that the GAD consisted of 22 girls and eight boys with a mean age of 10.11 ± 1.69. The other anxiety disorder group consisted of 23 girls and seven boys with a mean age of 10.22 ± 1.29. Twenty-six of the control group were female and 14 were male, with a median age of 9.95 (8–11.5 percentile). No significant differences were found when the groups were compared in terms of age and gender. The prevalence of previous psychiatric consultations was significantly higher in the GAD and other anxiety disorder groups than in the control group (*p* = 0.002). The income level and education level of the mothers in the control group were significantly higher than in the other two groups. No differences were found in other variables. Table S1 summarizes the sociodemographic data. In our study, 17 individuals in the “other anxiety disorders” group were diagnosed with other defined anxiety disorders: eight had separation anxiety disorder, three had social anxiety disorder, and two had a specific phobia.

When groups were compared in terms of scale scores, the GAD and other anxiety disorders group demonstrated significantly higher scores than the control group on the SCARED, ASI-3 total, physical and cognitive subscales, as well as on the ERC Emotional Variability/Negativity and ERC Emotion Regulation subscales (*p* < 0.001). The present study found no significant differences between GAD, other anxiety disorders, and the control group in terms of the ASI-3 social subscale and the BIS-S (Table 1). Adjusted comparisons were made by adding maternal education, family income, and maternal employment status to the analysis due to differences in sociodemographic data between groups. Covariate-adjusted analyses controlling for maternal education, family income, and maternal employment status yielded largely consistent results with the unadjusted comparisons: group differences remained significant for SCARED, ASI-3 total/physical, and ERC subscales (overall *p* < 0.001), whereas the adjusted group effect for BIS-S (overall *p* = 0.240) and ASI-3 social (overall *p* = 0.333) remained non-significant, suggesting that the null BIS-S and ASI-3 social finding was not explained by sociodemographic confounding. However, after adjusting for potential confounders, the ASI-3 Cognitive subscale was no longer significant between the two groups (overall *p* = 0.074) (Table 2).

Given the heterogeneity of the ‘other anxiety disorders’ group, we conducted sensitivity analyses by testing diagnosis-by-predictor interactions within the clinical sample (GAD vs. other anxiety disorders), using rank-based models adjusted for maternal education, family income, and maternal employment status. Notably, the BIS-S association with ASI-3 total and ASI-3 physical differed by diagnosis (interaction *p* < 0.05), supporting cautious interpretation of pooled ‘other anxiety disorders’ findings.

The relationship between impulsivity scores and other scales was evaluated within the anxiety disorders group. No significant relationship was found between impulsivity scores and other scales (SCARED, BIS-S, ASI-3, and ERC) in either group when evaluating the relationships between them.

The relationship between anxiety severity and anxiety sensitivity was evaluated in the anxiety disorders group. In the anxiety disorder group, a positive correlation was identified between the SCARED score and the total and physical scores of the ASI-3 (r = 0.619, *p* < 0.001, q = 0.021; r = 0.538, *p* = 0.002, q = 0.021). In contrast, no significant relationship was observed in the generalized anxiety disorder group.

The relationship between anxiety severity and Emotion Regulation Scale subscale scores was evaluated in the anxiety disorders group. In the other anxiety disorders group, a negative correlation was found between the ERC Emotion Regulation subscale and the SCARED (r = −0.480, *p* < 0.007, q = 0.049), though no significant relationship was found in the GAD. When the relationship between the ERC and the ASI-3 was evaluated in the anxiety disorders group, no significant relationship was found. The correlations between the scales are summarized in Table 3.

To formally test the hypothesized indirect mechanism, three mediation models were conducted within the clinical sample (n = 60) using bootstrap confidence intervals (5000 resamples). Across all models, anxiety sensitivity did not significantly mediate the association between emotion regulation indices (ERC) and impulsivity (BIS-S), as the indirect effects were non-significant and their 95% bootstrap CIs included zero (Model 1 indirect effect B = 0.002, 95% CI [−0.032, 0.056], *p* = 0.923; Model 2 B = −0.001, 95% CI [−0.026, 0.025], *p* = 0.939; Model 3 B = 0.010, 95% CI [−0.038, 0.064], *p* = 0.671). In contrast, ERC Variability/Negativity showed significant total and direct associations with impulsivity in Models 2–3 (Model 2: total effect B = 0.304, 95% CI [0.076, 0.558], *p* = 0.014; direct effect B = 0.305, 95% CI [0.076, 0.557], *p* = 0.014; Model 3: total effect B = 0.304, 95% CI [0.079, 0.570], *p* = 0.011; direct effect B = 0.294, 95% CI [0.068, 0.548], *p* = 0.013), whereas the model with ERC emotion regulation was not significant. Detailed estimates and path diagrams are provided in Table 4.

## 4. Discussion

This study evaluated impulsivity in childhood generalized anxiety disorder and other anxiety disorders, examining the relationship between impulsivity and anxiety sensitivity as well as emotion regulation. In the generalized anxiety disorder and other anxiety disorder group, the SCARED, ASI-3 total/physical, and ERC subscales were significantly higher than in the control group. However, there were no significant differences in impulsivity and ASI-3 social and cognitive dimension scores. No significant relationship was identified between impulsivity and other scale scores in either group of anxiety disorders. In the other anxiety disorders group, it was found that as the anxiety severity score increased, there was an increase in the total and physical subscales of the ASI-3, while there was a decrease in the ERC-emotion regulation subscale score. No significant relationship was found in the GAD group. According to the mediation model, anxiety sensitivity does not significantly mediate the relationship between emotion regulation and impulsivity. Emotional variability/negativity are directly and fully related to impulsivity.

In our study, no significant differences in impulsivity scores were found between the GAD, other anxiety disorders, and the control group among children. Studies indicate a strong positive correlation between anxiety and impulsivity in disorders involving impulse control and impulsivity [29,66]. The presence of anxiety in mood disorders has also been shown to increase impulsive behaviors, such as suicidal thoughts and attempts [67,68]. However, the relationship between anxiety disorders and impulsivity is controversial and has not been sufficiently clarified [53,69]. Few studies have examined the role of impulsivity in primary anxiety disorders [28,29,70]. A study comparing impulsivity in individuals with anxiety disorders (social anxiety disorder, panic disorder, and agoraphobia) and a healthy control group found that the case group was more impulsive than the control group in all investigated measurements [29]. It has been noted that impulsive behaviors increase as a result of the high arousal caused by anxiety. Another study assessed impulsivity in individuals with social phobia, agoraphobia, and specific phobia. It was noted that the impulsivity characteristics of the social phobia and agoraphobia groups were higher than those of the specific phobia and control groups [38], unpublished thesis). Similar results were found in another study where impulsivity was assessed using both a scale and neuropsychological measurements [71]. Studies have reported higher impulsivity in anxiety disorders than in the control group and found that emotional instability and negative affect mediate this difference [71,72]. Notably, these studies have been conducted in the adult age group. In both adults and adolescents, the amygdala is highlighted as a particularly important region in the development and maintenance of pathological anxiety [73]. The function and organization of the dorsolateral prefrontal cortex, a brain region implicated in anxiety, and its connections with other related structures, such as the amygdala, are critical for working memory, executive function, and the regulation of emotions, particularly anxiety. The prefrontal cortex (PFC), anterior cingulate cortex, and amygdala have been identified as the neural structures underlying impulse control [74]. Impulsivity is thought to follow a normative developmental trajectory from childhood to adulthood, guided by the maturation of the relevant brain structures [75]. Research indicates that between the ages of 6 and 12, there is a decrease in some aspects of impulsivity with the development of cognitive control and self-regulation [76]. During this period, PFC undergoes significant maturation in both gray and white matter [77,78]. and cognitive functions continue to develop [79]. Research indicates that the rise in impulsivity during adolescence is associated with the ongoing development of the PFC [80]. In adults, increased impulsivity is frequently linked to decreased thickness of the cerebral cortex in regions of the PFC [81,82,83]. Given that neurophysiological and prefrontal cortex development continues during childhood, there may be differences in our study results compared to those in adulthood. Additionally, impulsivity is recognized as a multidimensional concept [84,85]. It is also conceivable that different components of impulsivity in children and adults may be associated with psychopathology.

Our study indicated a potential link between emotional variability/negativity and impulsivity in individuals diagnosed with anxiety disorders. In cases of anxiety, difficulties in decision-making processes may occur during periods of intense negative emotional states [33]. It has been noted that executive functions may be impaired, and individuals may make impulsive decisions in an attempt to alleviate intense distress [33,47,86]. Emotional variability/negativity scores are reported to include emotional instability, a tendency toward anger outbursts, mood swings, emotional intensity, and difficulties regulating positive emotions [86]. Research has shown that the emotional variability/negativity subscale is linked to maladaptive strategies, and that as emotional variability increases, individuals tend to employ inappropriate coping methods [86,87,88,89]. Research evaluating the relationship between emotion regulation skills and impulsive behaviors has indicated that difficulties in emotion regulation may serve as a specific risk factor in the development and maintenance of impulsivity [45]. Research has demonstrated that emotional regulation capacity exhibits fluctuations throughout the lifespan, attaining a more stable state during middle adulthood [19,90]. Given the shared neuroanatomical underpinnings and developmental trajectories of impulsivity and emotion regulation, emotional variability or lability in children is likely to be associated with impulsivity. Upon the study’s conclusion, the emotional instability and negativity exhibited by the anxiety disorders group may have resulted in the children’s adoption of maladaptive coping mechanisms, characterized by impulsive behaviors and a lack of emotional regulation. In order to establish a direct relationship between emotional variability/negativity and impulsivity, it is recommended that the data be replicated in the future with larger samples.

Our study revealed that the total ASI-3 total score and physical subscale scores were significantly higher in the GAD and other anxiety disorders group compared to the control group. However, there was no difference in the social and cognitive subscale scores. Research indicates that total anxiety sensitivity does not differ significantly between anxiety symptoms and disorders [91,92]. Our study found no statistically significant differences in total GAD scores between the GAD and other anxiety disorders groups. It has been proposed that anxiety sensitivity may not be a specific risk factor for GAD; rather, a comparative analysis of the subdimensions across different anxiety disorders is recommended [92].

Anxiety sensitivity, a significant risk factor for emotional disorders, has been linked to conditions such as generalized anxiety disorder, major depressive disorder, post-traumatic stress disorder, and obsessive–compulsive disorder [90,93]. Research has indicated that children and adolescents with high anxiety sensitivity are more likely to develop anxiety-related psychopathology, including panic disorder, generalized anxiety disorder, social anxiety disorder, and separation anxiety [94,95]. Research has demonstrated that distinct dimensions of anxiety sensitivity are associated with various anxiety disorders [26,90,93,96,97]. A review of the extant literature reveals that social anxiety is predominantly associated with the social dimension, generalized anxiety with the cognitive dimension, and panic disorder with the physical dimension and cognitive dimension [96,98,99,100]. Consequently, the low prevalence of social anxiety and panic disorder in our study may have impeded the differentiation between anxiety disorders and the control group with respect to the social dimension and cognitive scores of the ASI-3. In the present study, it was also observed that as anxiety severity increased in the other anxiety disorders group, the anxiety sensitivity index total and physical dimension scores increased. In our study, it was known that most of the other anxiety disorders group had another defined anxiety disorder and separation anxiety disorder. The frequent presence of somatic complaints in childhood separation anxiety disorders may have led to differentiation from other anxiety disorders, which primarily focus on the physical dimension of anxiety sensitivity. Furthermore, since studies evaluating anxiety sensitivity have been conducted in adult samples, and to the best of our knowledge, there are no studies in the current literature evaluating the relationship between separation anxiety and anxiety sensitivity in children, it can be said that our findings may contribute to the field. Furthermore, given the adjusted analyses conducted with demographic variables in the other anxiety disorder group, it is essential to exercise caution when interpreting the findings of the other anxiety disorders group. It is recommended that the findings be re-evaluated in the future with a larger sample group. In our study, we observed that the significance of the cognitive dimension of the ASI-3 was lost in scale group comparisons that were adjusted for sociodemographic confounding factors. Given the potential impact of sociodemographic factors on the cognitive dimension of anxiety sensitivity, it is advised that future studies replicate the data by matching the demographic structure between groups.

It has been suggested that anxiety sensitivity may be a risk-reducing factor in the face of potential adversities [36,37] and may protect against impulsive behaviors [36], but this has not yet been clearly established [35]. A study evaluating the relationship between anxiety sensitivity and impulsivity in an adult sample [38] reported no significant relationship. A study evaluating anxiety sensitivity in a non-clinical sample indicated that individuals with high anxiety sensitivity traits showed a significantly lower tendency to take risks compared to individuals with lower anxiety sensitivity traits [101]. Another study has indicated that young people who exhibit high anxiety sensitivity and impulsivity engage in risky behaviors, such as substance use [102]. In our study, however, no relationship was found between the total ASI-3 score and its subscales, including the impulsivity score. To our knowledge, there are no other studies evaluating anxiety sensitivity and impulsivity in children. Regarding the relationship between anxiety sensitivity and impulsivity, which has not yet been clarified in the field, no relationship was found in our study.

Our study evaluated the mediating role of anxiety sensitivity in the relationship between emotion regulation and impulsivity. The study revealed that anxiety sensitivity does not play a substantial mediating role in the relationship between emotion regulation and impulsivity. Although anxiety sensitivity and emotion regulation are distinct transdiagnostic factors, some studies have identified interactions between them [20,21]. Individuals with high anxiety sensitivity tend to use maladaptive emotional regulation strategies to avoid negative emotional states [103,104]. Individuals may adopt avoidance or escape behaviors to prevent such experiences [105,106]. It has also been reported that children with high anxiety sensitivity are more likely to engage in avoidance behaviors [107]. Consequently, it may be possible that anxiety sensitivity and emotion regulation interact, that avoidance may be the primary emotion regulation strategy, and that anxiety sensitivity may not be a mediating model for impulsivity. It is recommended that the data be re-evaluated in the future with larger samples.

One of the study’s limitations is the relatively small number of participants, which may affect the generalizability of the findings. A notable restriction of the study pertains to the assessment of impulsivity, which was conducted exclusively on a cross-sectional basis through the utilization of a scale based on self-report. The employment of neurocognitive impulsivity measures was omitted, and the study’s scope was confined to mothers. Additionally, other anxiety disorders are evaluated under a single heading, in contrast to GAD. Due to the limited representation of individual non-GAD anxiety disorder subtypes, subtype-specific analyses were not conducted. Consequently, the findings pertaining to "other anxiety disorders" should be regarded as exploratory. Despite these important limitations, our study is, to our knowledge, the first to examine the relationship between impulsivity and anxiety sensitivity and emotion regulation in children with GAD and other anxiety disorders. Other strengths include the fact that impulsivity was assessed in a group of children with only anxiety disorders who did not have other psychiatric disorders, that adolescents were not included, and that children who were not using psychotropic medications were included. In the future, it is recommended that the data be validated with a larger sample group in which GAD and other anxiety disorders are evaluated separately. In addition, studies comparing each anxiety disorder within itself can be said to contribute to this field.

## 5. Conclusions

Consequently, our study found no significant differences in impulsivity scores between GAD, other anxiety disorders, and the control group. In the generalized anxiety disorder and other anxiety disorder groups, the total/physical and ERC subscales of the SCARED and ASI-3 were significantly higher than in the control group. Anxiety sensitivity was found not to significantly mediate the relationship between emotional variability/negativity and impulsivity; emotional variability/negativity was found to be directly and fully associated with impulsivity. The findings of the present study indicate that children diagnosed with anxiety disorders encounter heightened challenges in regulating their emotions in comparison to children without such diagnoses. Furthermore, the results suggest a direct correlation between emotional variability/negativity and impulsivity. This observation lends support to the notion that anxious children who are incapable of regulating their emotions may manifest maladaptive impulsive coping behaviors. In this context, the enhancement of emotion regulation skills in anxiety disorders may prove to be a pivotal factor in the efficacy of treatment and the maintenance of behavioral control. To summarize, given the intricate neurobiology of impulsivity, the potential for other as yet unknown mediating mechanisms to influence anxiety disorders through a variety of factors is a promising avenue for future research.

## Figures and Tables

**Table 1:** Comparison of scale scores of the groups.

	Generalized Anxiety Disorders Group *n* = 30	Other Anxiety Disorders Group *n* = 30	Control Group *n* = 40	*p*	Overall *p*
The Screen for Child Anxiety Related Emotional Disorders *	42.18 ± 10.95	35.33 ± 14.86	22.78 ± 10.47	<0.001 ^a^	<0.001 ^c^
Post hoc: Control group < GAD, Other Anxiety Disorders (Tamhane *p* < 0.05)					
Children’s Anxiety Sensitivity Index (ASI-3) score *	35.71 ± 5.95	34.40 ± 5.75	28.50 ± 5.84	<0.001 ^a^	<0.001 ^c^
Post hoc: Control group < GAD, Other Anxiety Disorders (Tukey *p* < 0.05)					
ASI-3 Physical subscale score *	24.46 ± 4.50	22.96 ± 5.13	19.22 ± 4.54	<0.001 ^a^	<0.001 ^c^
Post hoc: Control group < GAD, Other Anxiety Disorders (Tukey *p* < 0.05)					
ASI-3 Cognitive subscale score **	5 (4–7)	4 (4–6.25)	3 (3–4)	<0.001 ^b^	0.074 ^c^
ASI-3 Social subscale score **	6 (5–7)	7 (6–7)	6 (5–7)	0.103 ^b^	0.333 ^c^
Barrett Impulsivity Scale score **	30.50 (28–34)	29 (25–32.50)	27 (23–33)	0.082 ^b^	0.240 ^c^
Emotion Regulation Checklist/Emotion Regulation Subscale Score *	19.93 ± 4.46	20.97 ± 4.50	16.15 ± 3.30	<0.001 ^a^	<0.001 ^c^
Post hoc: Control group < GAD, Other Anxiety Disorders (Tukey *p* < 0.05)					
Emotion Regulation Checklist/Emotional variability/negativity subscale score *	28.82 ± 5.34	27.30 ± 4.99	23.68 ± 4.67	<0.001 ^a^	0.001 ^c^

^a^ One-way ANOVA test, ^b^ Kruskal–Wallis test, ^c^: ANCOVA, *: Mean ± Standard Deviation, **: (25–75 percentiles), GAD: Generalized Anxiety Disorders, ASI-3: Children’s Anxiety Sensitivity Index.

**Table 2:** Covariate-adjusted group comparisons of scale scores (control as reference).

Outcome	Model	Overall *p* (Group)	GAD vs. Control: β (95% CI); *p*	Other AD vs. Control: β (95% CI); *p*
SCARED total (SCARED)	ANCOVA (robust SE)	<0.001	20.39 (14.88, 25.90); *p* ≤ 0.001	13.50 (5.56, 21.43); *p* ≤ 0.001
ASI-3 total	ANCOVA (robust SE)	<0.001	6.93 (3.91, 9.95); *p* ≤ 0.001	5.42 (1.82, 9.03); *p* = 0.003
ASI-3 Physical	ANCOVA (robust SE)	<0.001	5.00 (2.64, 7.36); *p* ≤ 0.001	3.36 (0.32, 6.40); *p* = 0.030
ASI-3 Cognitive	Median regression (q = 0.5)	0.074	1.00 (0.04, 1.96); *p* = 0.043	1.00 (−0.02, 2.02); *p* = 0.057
ASI-3 Social	Median regression (q = 0.5)	0.333	0.21 (−0.63, 1.04); *p* = 0.627	0.67 (−0.22, 1.55); *p* = 0.145
BIS-S total (BIS-S)	Median regression (q = 0.5)	0.240	3.00 (−0.52, 6.52); *p* = 0.098	1.00 (−2.75, 4.75); *p* = 0.602
ERC/Emotion Regulation	ANCOVA (robust SE)	<0.001	3.41 (1.32, 5.51); *p* = 0.001	4.42 (2.15, 6.69); *p* ≤ 0.001
ERC/Emotional variability-negativity	ANCOVA (robust SE)	0.001	4.71 (2.09, 7.34); *p* ≤ 0.001	3.04 (0.62, 5.46); *p* = 0.014

AD = anxiety disorders; GAD = generalized anxiety disorder; BIS-S = Barrett Impulsivity Scale; SCARED = The Screen for Child Anxiety Related Emotional Disorders; ASI-3 = Children’s Anxiety Sensitivity Index; ERC = Emotion Regulation Checklist.

**Table 3:** Relationship of scores in the case group with other scales.

	Groups		1	2	3	4
1. Childhood Anxiety Screening Scale score	GAD	r				
		*p*/q				
	Other Anxiety Disorders	r				
		*p*/q				
2. Emotion Regulation Scale/Emotional variability/negativity subscale score	GAD	r				
		*p*/q	0.101/0.231			
	Other Anxiety Disorders	r				
		*p*/q	0.287/0.430			
3. Emotion Regulation Scale/Emotion Regulation subscale Score	GAD	r	−0.391			
		*p*/q	0.033/0.196			
	Other Anxiety Disorders	r	−0.480			
		*p*/q	0.007/0.049			
4. Barrett Impulsivity Total Score	GAD	r	−0.365	0.364		
		*p*/q	0.047/0.196	0.048/0.196	0.358/0.470	
	Other Anxiety Disorders	r				
		*p*/q	0.631/0.665	0.140/0.371	0.429/0.528	
5. ASI-3 Total Score	GAD	r	0.353			
		*p*/q	0.056/0.196	0.146/0.256	0.698/0.797	0.121/0.231
	Other Anxiety Disorders	r	0.619			
		*p*/q	<0.001/0.021	0.250/0.404	0.077/0.294	0.158/0.371
6. ASI-3 Social subscale	GAD	r				
		*p*/q	0.721 */0.797	0.267 */0.374	0.933 */0.936	0.098 */0.231
	Other Anxiety Disorders	r				
		*p*/q	0.453 */0.528	0.212 */0.380	0.217 */0.380	0.840 */0.840
7. ASI-3 Physical subscale	GAD	r				
		*p*/q	0.119/0.231	0.412/0.509	0.936/0.936	0.252/0.374
	Other Anxiety Disorders	r	0.538			
		*p*/q	0.002/0.021	0.202/0.380	0.159/0.371	0.067/0.294
8. ASI-3 Cognitive subscale	GAD	r	0.529	−0.483		
		*p*/q	0.003 */0.063	0.007 */0.073	0.115 */0.231	0.225 */0.363
	Other Anxiety Disorders	r				
		*p*/q	0.084 */0.294	0.345 */0.453	0.316 */0.442	0.633 */0.665

Multiple comparisons in Table 3 were controlled using the Benjamini–Hochberg false discovery rate (FDR) procedure within each diagnostic group (m = 22 tests per group); q < 0.05 was considered significant, r: correlation coefficient, * Spearman correlation test, GAD: Generalized Anxiety Disorder, ASI-3: Children’s Anxiety Sensitivity Index.

**Table 4:** Mediation analysis results and path diagrams.

Effect	Model 1 X: ERC Emotion Regulation M: ASI-3 Total Y: BIS-S Total	Model 2 X: ERC Lability/Negativity M: ASI-3 Total Y: BIS-S Total	Model 3 X: ERC Lability/Negativity M: ASI-3 Cognitive Y: BIS-S Total
a (X→M)	0.149 (0.164); *p* = 0.368; 95% CI [−0.157, 0.483]	−0.029 (0.116); *p* = 0.811; 95% CI [−0.261, 0.207]	−0.051 (0.043); *p* = 0.226; 95% CI [−0.124, 0.043]
b (M→Y)	0.014 (0.094); *p* = 0.885; 95% CI [−0.163, 0.209]	0.032 (0.094); *p* = 0.732; 95% CI [−0.149, 0.212]	−0.197 (0.318); *p* = 0.545; 95% CI [−0.783, 0.475]
c (total X→Y)	0.133 (0.126); *p* = 0.295; 95% CI [−0.123, 0.385]	0.304 * (0.121); *p* = 0.014; 95% CI [0.076, 0.558]	0.304 * (0.127); *p* = 0.011; 95% CI [0.079, 0.570]
c’ (direct X→Y)	0.131 (0.130); *p* = 0.312; 95% CI [−0.139, 0.383]	0.305 * (0.122); *p* = 0.014; 95% CI [0.076, 0.557]	0.294 * (0.125); *p* = 0.013; 95% CI [0.068, 0.548]
Indirect (a × b)	0.002 (0.021); *p* = 0.923; 95% CI [−0.032, 0.056]	−0.001 (0.012); *p* = 0.939; 95% CI [−0.026, 0.025]	0.010 (0.024); *p* = 0.671; 95% CI [−0.038, 0.064]

ERC = Emotion Regulation Checklist; ASI-3 = Anxiety Sensitivity Index. * = *p* < 0.05; The indirect effect is a × b; c is the total effect; and c’ is the direct effect that controls for the mediating variable. → path.

## Data Availability

The data presented in this study are not publicly available due to ethical restrictions; however, they can be provided upon request from the corresponding author.

## Supplementary Materials

The following supporting information can be downloaded at: https://www.mdpi.com/article/10.3390/children13030439/s1, Table S1: Sociodemographic Data.
