# Supplementary material for: The Link Between Emotional Regulation and Impulsivity in Childhood Anxiety Disorder
Source: Children (Basel). 2026 Mar 23;13(3):439. doi: 10.3390/children13030439 (PMC13038219; doi:10.3390/children13030439)
Supplement: Supplementary file 1 [file children-13-00439-s001.zip › children-4144640-supplementary.pdf]

Table S1: Sociodemographic Data.

		Generalized Anxiety Disorder group $n = 30$	Other Anxiety Disorders group $n = 30$	Control group $n = 40$	p
Gender	Female	23	22	26	0.536 [a]
	Male	7	8	14	
Age	Child's age (years)	10.22 ± 1.29 **	10.11 ± 1.69 **	9.95 (8 – 11.5) *	0.443 [c]
	Mother's age (years)	39.07 ± 4.83 **	38.67 ± 5.96 **	39.38 ± 4.07 **	0.838 [b]
	Father's age (years)	43.27 ± 4.97 **	42.40 ± 6.25 **	42.48 ± 4.09 **	0.758 [b]
Children's school situation	Elementary	14	12	23	0.334 [a]
	Middle school	16	18	17	
Children's previous psychiatric visits	Yes	11	14	4	0.002 [a]
	None	19	16	36	
Mother's employment status	Working	18	14	35	0.001 [a]
	Not working	12	16	5	
Father's employment status	Working	29	28	38	0.839 [a]
	Not working	1	2	2	
Mother's educational status	Elementary/middle school	5	10	3	0.019 [a]
	High school	9	11	10	
	College/University	16	9	27	
Father's educational status	Elementary/middle school	5	8	2	0, 154 [a]
	High school	11	8	15	
	College/University	14	14	23	
Family income status	Low	7	7	4	0.025 [a]
	Middle	11	15	10	
	High	12	8	26	

[a] Pearson Chi Square Test [b] One Way ANOVA Test [c] Kruskal Wallis Test
*: (25-75 percentile) **: Mean ± Standard Deviation
